# Dose‐Adjusted Treatment With 7‐Day Venetoclax Plus Azacitidine or Decitabine in Patients With Acute Myeloid Leukaemia

**DOI:** 10.1111/ejh.70279

**Published:** 2026-07-30

**Authors:** Laurenz Steiner, Lina S. Silva‐Bermudez, Mahmoud Ghazal, Mohammed Abba, Catharina Gerhards, Georgia Metzgeroth, Alice Fabarius, Stefan Klein, Sebastian Kreil, Nanni Schmitt, Patrick Wuchter, Wolf‐Karsten Hofmann, Alexander Streuer

**Affiliations:** ^1^ Department of Haematology and Oncology, Medical Faculty Mannheim Heidelberg University Mannheim Germany; ^2^ German Red Cross Blood Service Baden‐Württemberg‐Hessen, Medical Faculty Mannheim, Institute of Transfusion Medicine and Immunology Heidelberg University Mannheim Germany; ^3^ Medical Faculty Mannheim, Institute for Clinical Chemistry Heidelberg University Mannheim Germany

**Keywords:** acute myeloid leukaemia, dose‐adjusted treatment, hypomethylating agents, venetoclax

## Abstract

Acute myeloid leukaemia (AML) in older or unfit patients is commonly treated with hypomethylating agents (HMA) plus venetoclax (VEN), but prolonged VEN exposure often causes substantial haematological toxicity. We retrospectively analysed 61 elderly AML patients treated with HMA + VEN for 7 (7d), 14 (14d) or > 14 days (> 14d) per cycle. Best overall response rate was 76% and did not differ significantly between schedules (*p* = 0.38). Median overall survival (OS) was numerically longer with 7d (28.4 months) than with 14d (11.4 months) or > 14d (6.7 months), although unadjusted differences were not significant (*p* = 0.23). At cycle level, the 7d schedule was associated with fewer infections and more complication‐free cycles than both longer schedules, while bleeding events were similar. Grade 4 cytopenias and platelet transfusion requirements were also lower with 7d. In an adjusted Cox model, prior HMA exposure, non‐favourable ELN 2024 risk and age ≥ 75 years were associated with inferior OS, whereas the 7d schedule remained associated with improved OS versus > 14d (HR 0.33, *p* = 0.03). Higher recent grade 4 neutropenia burden was independently associated with inferior OS. These findings suggest that abbreviated VEN exposure may improve tolerability without clear loss of efficacy in AML patients ineligible for intensive chemotherapy.

## 1 Introduction

Acute myeloid leukaemia (AML) predominantly affects older adults, who often have substantial comorbidity, impaired fitness and limited eligibility for intensive chemotherapy or allogeneic haematopoietic stem cell transplantation (allo‐HSCT). In this setting, hypomethylating agents (HMA) combined with venetoclax (VEN), a selective B‐cell leukaemia/lymphoma 2 (BCL2) inhibitor, have become a standard‐of‐care low‐intensity treatment approach after demonstrating superior survival compared with azacitidine (AZA) alone [1, 2]. Real‐world data further support the feasibility of VEN‐based low‐intensity regimens in very old patients [3].

The HMA most commonly used in AML are AZA and decitabine (DEC). Standard treatment schedules combine AZA for 7 days (7d) or DEC for 5 days with VEN for up to 28 days in 28‐day cycles [2, 4]. However, prolonged VEN exposure is frequently associated with substantial haematological toxicity, particularly prolonged neutropenia, thrombocytopenia, infectious complications, treatment delays and cycle modifications [5]. These toxicities are especially relevant in older or frail patients, in whom treatment tolerability may critically determine the ability to continue therapy.

Accordingly, there is increasing interest in abbreviated VEN schedules. Recent observational studies evaluating 7d or 14‐day (14d) VEN exposure in combination with AZA have suggested that shorter schedules may retain anti‐leukaemic efficacy while reducing toxicity [6, 7, 8]. Nevertheless, evidence remains limited, and concerns persist that shortening VEN exposure could compromise disease control in biologically high‐risk AML, including subgroups with reduced VEN sensitivity [9, 10]. Against this background, we evaluated the feasibility of a shortened 7d VEN schedule combined with standard‐dose AZA or DEC in a retrospective real‐world AML cohort. We compared response, survival, complications, cytopenias and transfusion burden across 7d, 14d and > 14d VEN schedules, and explored whether time‐varying neutropenia burden contributed to observed survival differences.

## 2 Patients and Methods

We retrospectively analysed adults with AML treated at our institution between 2018 and 2025 who were considered ineligible for intensive chemotherapy or at high risk for treatment‐related complications and who received VEN in combination with AZA and/or DEC in first‐line or later‐line treatment. AZA was administered at 75 mg/m^2^ subcutaneously for 7d (5 + 2 schedule), corresponding to Days 1–5 and 8–9 of each nominal 28‐day cycle. DEC was administered at 20 mg/m^2^ intravenously on Days 1–5. VEN exposure per cycle was determined by the treating physician according to clinical factors, including frailty, cytopenia, comorbidities and prior tolerance.

Patients were assigned to one of three VEN schedule groups according to their predominant VEN exposure across documented treatment cycles: 7d, 14d or 15–28 days (> 14d). In all patients, VEN was initiated on Day 1 of the HMA‐based treatment cycle and continued until the respective scheduled final VEN day within the planned cycle. Standard VEN dose was 400 mg once daily, with dose reduction to 100 mg once daily during concomitant azole therapy or when clinically indicated. In the 7d cohort, seven patients received 7d VEN from cycle 1, whereas seven were switched from longer exposure after a median of 2 cycles (range 1–6). In 13 of 14 patients, the 7d schedule accounted for > 50% of all cycles; in one patient, 7d accounted for 50%. To address possible bias introduced by the assignment of patients to cohorts based on predominant VEN exposure, a sensitivity analysis classifying patients as receiving exclusively 7d of VEN in all documented cycle schedules was performed for the ‘Always‐7d’ cohort. Patients with any other documented VEN exposure were assigned to the corresponding longer‐schedule group.

Response was assessed according to the 2022 European LeukemiaNet (ELN) response criteria for AML [11]. Best overall response was defined as the deepest response achieved after treatment initiation and before relapse or progression, death or initiation of subsequent anti‐leukaemic therapy, based on all available bone marrow and peripheral blood assessments. Overall response rate (ORR) was defined as complete remission (CR), CR with incomplete haematological recovery (CRi), CR with partial haematological recovery (CRh) or morphologic leukaemia‐free state (MLFS).

Treatment‐related complications, including infections, bleeding events, tumour lysis syndrome and other adverse events, were retrospectively annotated. Haematological toxicity was assessed using platelet counts and absolute neutrophil counts (ANCs), together with red blood cell (RBC) and platelet transfusion requirements, according to Common Terminology Criteria for Adverse Events (CTCAE) definitions [12]. Measurable residual disease (MRD) was assessed by real‐time quantitative polymerase chain reaction on bone marrow aspirates obtained during therapy; analyses were performed locally or at external accredited laboratories. Molecular data were derived from available next‐generation sequencing and PCR‐based AML panels. Genetic risk at diagnosis was assigned according to ELN 2022 and ELN 2024 criteria [11, 13].

### 2.1 Statistical Analysis

Categorical variables are reported as counts and percentages, and continuous variables as medians with ranges. Group comparisons used Fisher's exact test or chi‐squared test for categorical variables, as appropriate, and Kruskal–Wallis tests for overall continuous‐variable comparisons, with Wilcoxon rank‐sum tests for pairwise comparisons when indicated. Overall survival (OS) and time to permanent VEN discontinuation were estimated by the Kaplan–Meier method and compared using the log‐rank test. Pairwise survival comparisons were performed when indicated.

Associations with OS were explored using univariable Cox proportional hazards regression for prespecified baseline and treatment‐related covariates. Clinically relevant variables were then entered into multivariable Cox models. Hazard ratios (HRs) are reported with 95% confidence intervals (CIs). Baseline imbalances between treatment groups were assessed using standardised mean differences (SMDs), with values > 0.2 considered potentially meaningful.

To account for time‐varying treatment‐related cytopenia, we performed a time‐dependent Cox regression with start‐stop specification using rolling 30‐day grade 4 neutropenia burden. Grade 4 neutropenia was defined as ANC < 0.5 × 10^9^/L. ANC measurements were modelled as piecewise constant between sampling time points using the last observation carried forward. At each time point, the proportion of days with grade 4 neutropenia during the preceding 30 days was calculated as a moving‐window exposure variable (range 0–1). Thus, at each event time, the recent neutropenia burden of the patient experiencing the event was compared with that of all patients still at risk at the same follow‐up time. This approach accounts for irregular laboratory sampling and reduces fixed‐time and immortal‐time bias. An analogous rolling thrombocytopenia model was not prioritised because platelet counts may be directly modified by transfusion support and are therefore less readily interpretable as a biological time‐varying exposure in this setting.

All tests were two‐sided, and *p* < 0.05 was considered statistically significant. Analyses were performed using R version 4.4.3 (R Foundation for Statistical Computing, Vienna, Austria) and GraphPad Prism version 10.4.1.

## 3 Results

### 3.1 Broadly Comparable Baseline Risk Profiles Across VEN Exposure Cohorts

The study included 61 adults with AML (61% male), aged 43–86 years (mean 72.9 years). Twenty‐nine patients received VEN for > 14d, 18 for 14d and 14 for 7d per cycle (Table 1). In the > 14d cohort, 16 patients received AZA and 13 DEC; in the 14d cohort, 11 received AZA and 7 DEC and in the 7d cohort, 9 received AZA and 5 DEC. Schedule adaptations were frequent: VEN treatment days were reduced compared with cycle 1 in 31/61 patients (50.8%), 25/61 (41.0%) received a 7d schedule at least once, and prolonged intervals between VEN‐containing cycles occurred in 33/61 (54.1%) and 20/61 (32.8%) patients for intervals > 42 and > 56 days, respectively. VEN dose adjustments were documented in 28/61 patients (45.9%), predominantly in the context of concomitant azole therapy. During follow‐up, 40 deaths were recorded. Allo‐HSCT was performed in 10/61 patients (16%); among transplanted patients, four deaths occurred (> 14d, *n* = 2; 14d, *n* = 2; 7d, *n* = 0).

**TABLE 1:** Baseline and treatment characteristics.

Parameters	> 14d cohort (*n* = 29)	14d cohort (*n* = 18)	7d cohort (*n* = 14)	*p*	SMD (> 14 vs. 7 days)
Age (years) median [range]	72 [43–84]	73 [55–85]	78.5 [63–86]	0.18	−0.52
Male, *n* (%)	19 (66%)	10 (56%)	8 (57%)	0.79	
ECOG, *n* (%)				0.23	−0.39
ECOG 0–1, *n* (%)	16 (55%)	12 (67%)	5 (36%)		
ECOG 2–3, *n* (%)	13 (45%)	6 (33%)	9 (64%)		
Median BM blasts, % [range]	50 [2–90]	21 [0–90]	20 [3–90]	**0.027**	0.58
ANC (×10^9^/L) [range]	1.51 [0.24–42.33]	1.04 [0.04–28.74]	0.60 [0.08–3.83]	0.12	0.66
Haemoglobin (g/L) [range]	88 [72–149]	91 [67–117]	79 [50–133]	0.09	0.48
Platelets (×10^9^/L) [range]	56 [6–699]	41.5 [6–209]	53 [13–175]	0.87	0.23
Bilirubin (μmol/L) [range]	14.0 [6.3–44.5]	10.4 [5.0–24.1]	15.4 [6.5–84.3]	**0.04**	
Creatinine (μmol/L) [range]	96.4 [48.6–189.2]	88.4 [55.7–597.6]	77.8 [53.0–134.4]	0.18	
Median cycles given, *n* [range]	2 [1–14]	3 [1–8]	5 [1–20]	**0.03**	
Cytogenetics, *n* (%)^a^					
Normal/favourable					
Diploid	14 (48%)	8 (44%)	6 (43%)	1	
Diploid, −Y	1 (3%)	0 (0%)	1 (7%)	0.72	
t(8;21)	1 (3%)	0 (0%)	0 (0%)	1	
Other intermediate	4 (14%)	3 (17%)	4 (29%)	0.53	
Adverse					
−5/5q−	4 (14%)	4 (22%)	1 (7%)	0.59	
−7/7q−	5 (17%)	4 (22%)	3 (21%)	0.92	
−7/7q‐, −5	2 (7%)	2 (11%)	1 (7%)	0.85	
−17/17q−	1 (3%)	1 (6%)	1 (7%)	1	
−3/3q−	0 (0%)	1 (6%)	0 (0%)	0.53	
Complex karyotype	4 (14%)	2 (11%)	1 (7%)	1	
t(3;8)(q26;q24)	1 (3%)	0 (0%)	0 (0%)	1	
Molecular genetics, *n* (%)					
*IDH1/2*	8 (28%)	4 (22%)	4 (29%)	0.87	
*DNMT3A*	4 (14%)	3 (17%)	3 (21%)	0.90	
*SRSF2*	6 (21%)	2 (11%)	3 (21%)	0.75	
*ASXL1*	2 (7%)	4 (22%)	4 (29%)	0.14	
*TET2*	5 (17%)	2 (11%)	4 (29%)	0.43	
ELN 2022 risk category, *n* (%)					
Favourable	2 (7%)	2 (11%)	3 (21%)	0.75	
Intermediate	9 (31%)	5 (28%)	4 (29%)		
Adverse	18 (62%)	11 (61%)	7 (50%)		
ELN 2024 risk category, *n* (%)					
Favourable	14 (48%)	15 (83%)	10 (71%)	0.07	
Intermediate	12 (41%)	1 (6%)	3 (21%)		
Adverse	3 (10%)	2 (11%)	1 (7%)		
Non‐favourable (intermediate + adverse)	15 (52%)	3 (17%)	4 (29%)	0.20	0.47
AML type and prior therapy, *n* (%)					
AML‐MR	15 (52%)	11 (61%)	7 (50%)	0.80	
Previous HMA therapy	5 (17%)	9 (50%)	4 (29%)	0.06	0.27
Transplanted	4 (14%)	5 (28%)	1 (7%)	0.30	

*Note:* Bold values indicate statistical significance *p* < 0.05.

Abbreviations: 7d, 7 days; 14d, 14 days; ANC, absolute neutrophil count; AML, acute myeloid leukaemia; AML‐MR, acute myeloid leukaemia with myelodysplasia‐related changes; BM, bone marrow; d, day; ECOG, Eastern Cooperative Oncology Group; ELN, European LeukemiaNet; HMA, hypomethylating agent; SMD, standardised mean difference.

^a^ Selected cytogenetic features are not mutually exclusive.

Baseline characteristics were similar in several respects, but clinically relevant imbalances were present in selected variables, particularly between the 7d and > 14d cohorts. Median age did not differ significantly (*p* = 0.18). Eastern Cooperative Oncology Group (ECOG) performance status, ELN 2022 risk category, cytogenetic distributions and AML subtype were similar between groups. ELN 2024 risk showed a numerical imbalance, with a higher proportion of non‐favourable disease in the > 14d cohort than in the 7d cohort (52% vs. 29%; global *p* = 0.07; SMD 0.47). Median bone marrow blast percentage differed across groups (*p* = 0.03), and prior HMA exposure was numerically more frequent in the 14d cohort (*p* = 0.06). SMD suggested a relevant imbalance between the 7d and > 14d cohorts for age, baseline neutrophil count, bone marrow blasts and ELN 2024 risk. These variables were considered in adjusted survival analyses.

All patients received VEN 400 mg daily or the equivalent azole‐adjusted dose. Only one patient received granulocyte colony‐stimulating factor for treatment‐associated neutropenia.

### 3.2 Response Rates Were Comparable, and Time on Treatment Was Longest in the 7d Cohort

Among response‐evaluable patients, the ORR was 76% (37/49). ORR did not differ significantly between schedules: 68% (17/25) in the > 14d cohort, 77% (10/13) in the 14d cohort and 91% (10/11) in the 7d cohort (*p* = 0.38; Figure 1A).

**FIGURE 1:**
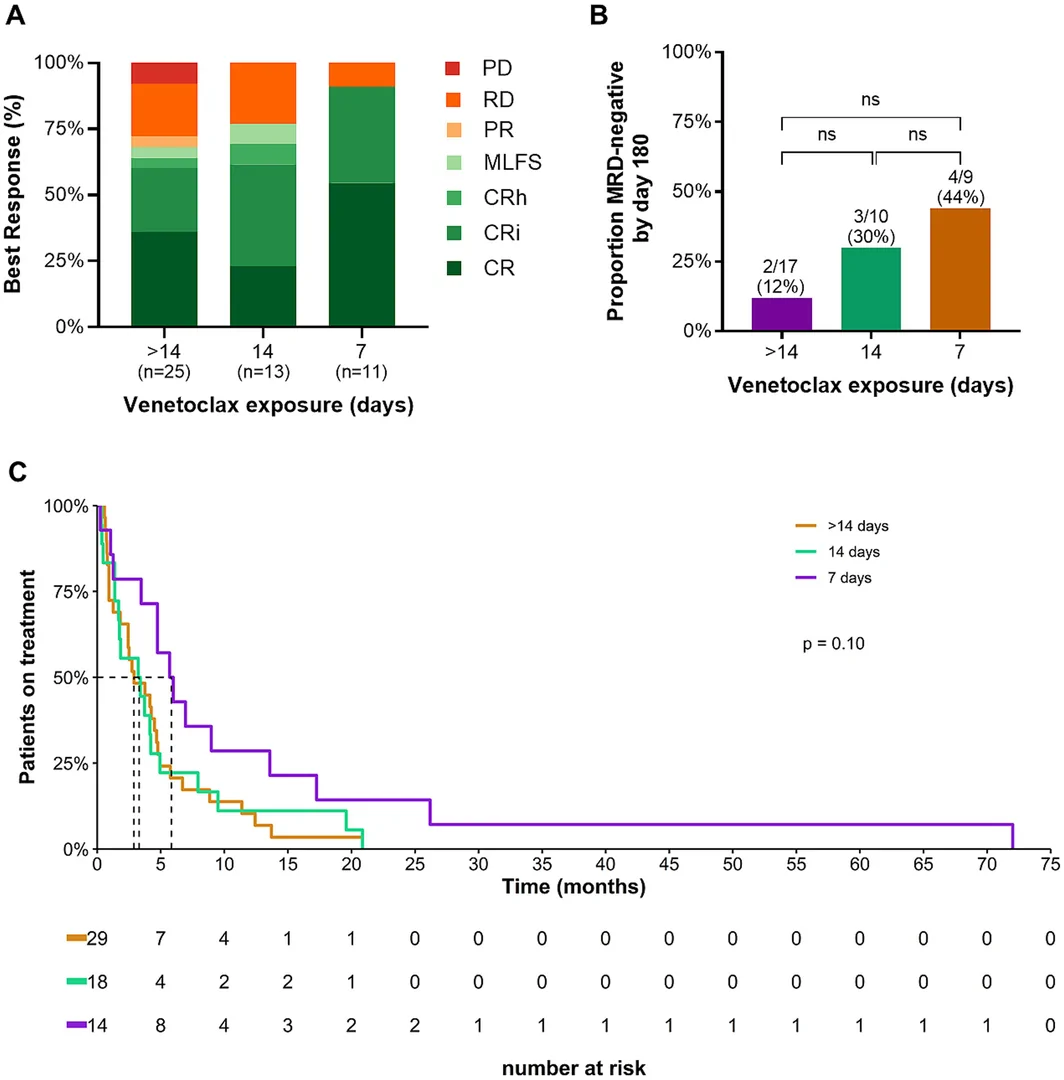
Overview of response, MRD and time to permanent venetoclax discontinuation. (A) Proportion of best responses among evaluable patients, stratified by per‐cycle venetoclax exposure (> 14, 14 and 7 days). CR, complete remission; CRi, complete remission with incomplete haematological recovery; CRh, CR with partial haematological recovery; MLFS, morphologic leukaemia‐free state; PR, partial remission; RD, refractory disease; PD, progressive disease. (B) Proportion of patients achieving minimal residual disease (MRD) negativity by Day 180 among those with ≥ 1 MRD assessment within 180 days after treatment initiation, stratified by per‐cycle venetoclax exposure. Bars indicate the fraction of MRD‐negative patients in each group; horizontal brackets show pairwise comparisons (Fisher's exact test), with non‐significant results labelled ‘ns’. (C) Kaplan–Meier curves of time to permanent venetoclax discontinuation according to schedule (> 14, 14 or 7 days per cycle). Time was measured from first VEN dose until discontinuation.

MRD negativity among patients with available MRD assessment by day 180 was also similar across groups. Rates were 44% (4/9) in the 7d cohort, 30% (3/10) in the 14d cohort and 12% (2/17) in the > 14d cohort; none of the pairwise comparisons reached statistical significance (Figure 1B).

Time to permanent VEN discontinuation differed numerically across schedules (Figure 1C). Median time on treatment was 2.9 months (range 0.6–20.9) in the > 14d cohort, 3.3 months (range 0.3–20.9) in the 14d cohort and 5.8 months (range 0.2–72.0) in the 7d cohort. The overall comparison showed a non‐significant trend (*p* = 0.10). In pairwise analyses, time on treatment was longer in the 7d cohort than in the > 14d cohort (*p* = 0.03), whereas 7d versus 14d (*p* = 0.10) and 14d versus > 14d (*p* = 0.97) were not significantly different.

Unadjusted OS did not differ significantly across the three predominant VEN schedule groups (*p* = 0.23; Figure 2A). Median OS was 6.7 months in the > 14d cohort, 11.4 months in the 14d cohort and 28.4 months in the 7d cohort. Pairwise log‐rank testing showed a non‐significant trend towards longer OS for 7d versus > 14d (*p* = 0.07), whereas 7d versus 14d was not significant (*p* = 0.33). A sensitivity analysis was performed, classifying patients who received 7 days of VEN in all documented cycle schedules as the Always‐7d group. Here, OS likewise did not differ significantly between groups (*p* = 0.53; Figure 2B), with median OS of 8.7 months in the > 14d group, 9.6 months in the 14d group and 18.2 months in the Always‐7d group (all pairwise comparisons were non‐significant).

**FIGURE 2:**
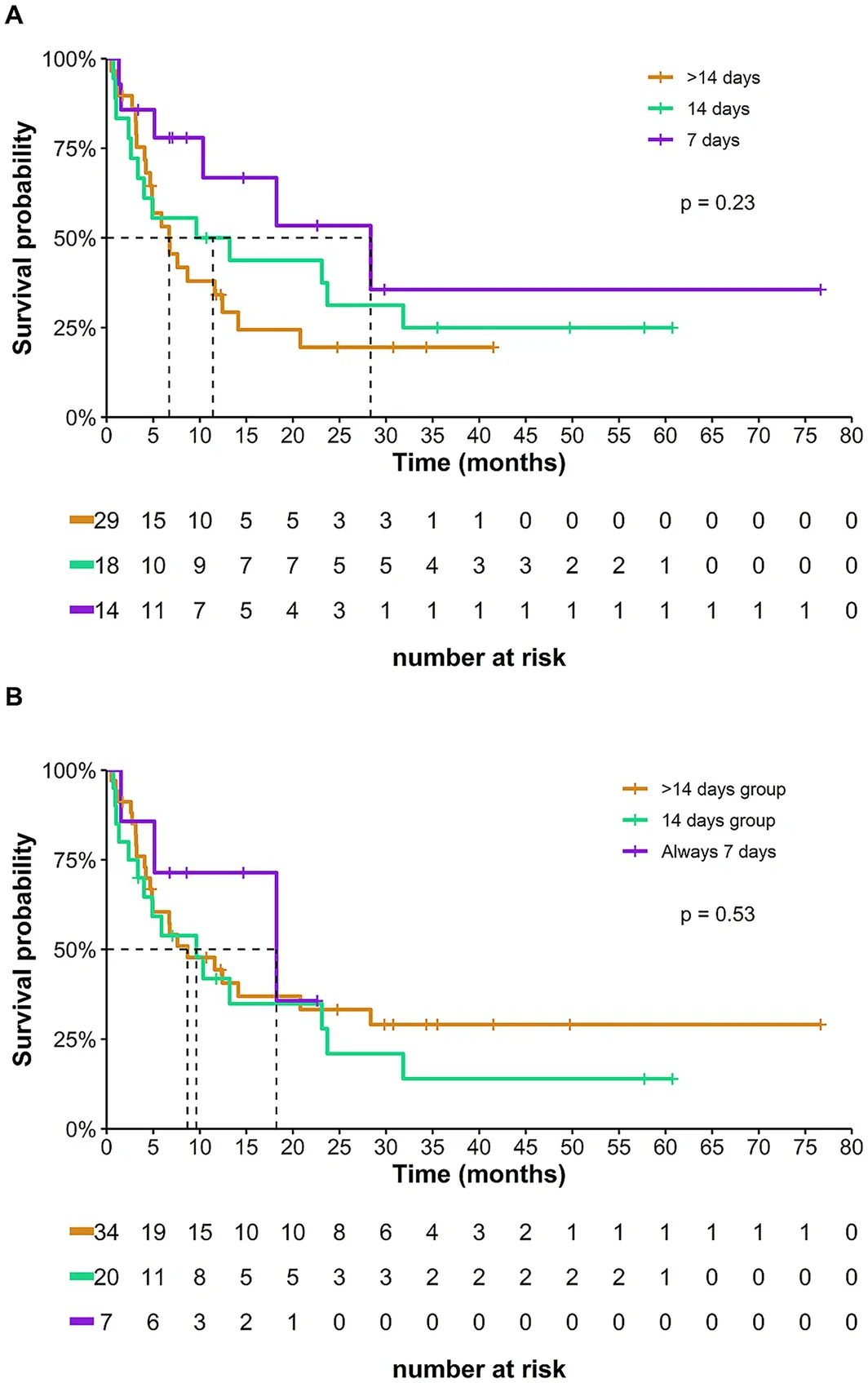
Overall survival by venetoclax schedule. Kaplan–Meier curves of overall survival according to venetoclax exposure. (A) Predominant per‐cycle venetoclax schedule: > 14 (*n* = 29) (14d), 14d (*n* = 18) and 7 days (7d) (*n* = 14). (B) Always‐7d sensitivity analysis: > 14d group (*n* = 34), 14d group (*n* = 20) and patients receiving 7 days of venetoclax in all documented cycles (Always 7d; *n* = 7). Numbers at risk are shown below each plot.

To address possible selection bias, an exploratory post‐remission analysis was performed. Here, 29/37 responders had documented post‐remission VEN‐containing cycles. The predominant post‐remission VEN schedule was 7 days in 9 patients, 14d in 8 patients and > 14d in 12 patients. OS from first remission did not differ significantly between post‐remission schedule groups (global log‐rank *p* = 0.48; Figure S1).

### 3.3 Shortened VEN Schedules Are Associated With Fewer Complications, Less Severe Cytopenias and Lower Platelet Transfusion Requirements

Cycle‐level adverse‐event analyses included 97 cycles in the 7d cohort, 59 in the 14d cohort and 103 in the > 14d cohort. The 7d schedule showed the most favourable descriptive complication profile. Infections occurred in 17/97 cycles (17.5%) in the 7d cohort, compared with 19/59 (32.2%) in the 14d cohort and 36/103 (35.0%) in the > 14d cohort. Infection rates were lower with 7d than with 14d (*p* = 0.035) and > 14d (*p* = 0.005). Conversely, complication‐free cycles were more frequent with 7d (78/97, 80.4%) than with 14d (37/59, 62.7%; *p* = 0.015) or > 14d (62/103, 60.2%; *p* = 0.002). Bleeding events were uncommon overall (2.1%–10.2% of cycles) and did not differ significantly between schedules. TLS and other adverse events were rare (Figure 3A). In the strict Always‐7d sensitivity analysis, infection‐associated cycles remained less frequent with Always 7d (4/43, 9.3%) than with 14d (21/72, 29.2%; *p* = 0.012) or > 14d (47/144, 32.6%; *p* = 0.003) and complication‐free cycles were more frequent (38/43, 88.4%) than with 14d (49/72, 68.1%; *p* = 0.014) or > 14d (90/144, 62.5%; *p* = 0.001).

**FIGURE 3:**
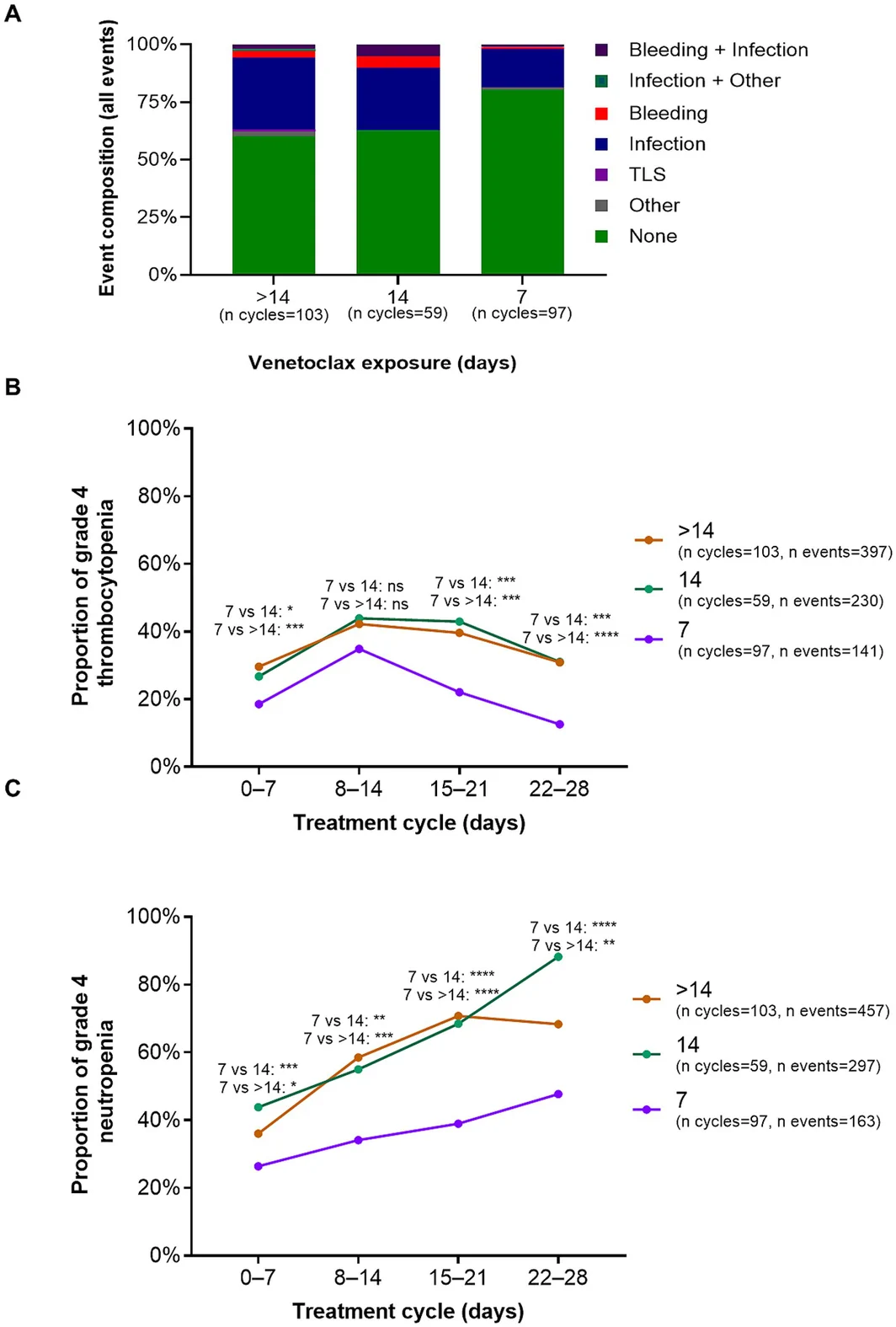
Treatment‐related adverse events. (A) Cycle‐level complications by venetoclax schedule. Stacked bars show the mutually exclusive distribution of complications per treatment cycle. Each cycle was assigned to one of the following categories: None, infection, bleeding, tumour lysis syndrome (TLS), other, infection + other or bleeding + infection. (B) Proportion of grade 4 thrombocytopenia in weekly intervals during Days 0–28 of the treatment cycle. (C) Proportion of grade 4 neutropenia in weekly intervals during Days 0–28 of treatment. *p* values for comparisons are indicated as *p < 0.05, **p < 0.01, ***p < 0.001, ****p < 0.0001.

Grade 4 thrombocytopenia (platelets < 25 × 10^9^/L) was less frequent in the 7d cohort than in the > 14d cohort in Weeks 1, 3 and 4 (all *p* < 0.001), whereas Week 2 did not differ significantly. Compared with the 14d cohort, the 7d cohort showed lower rates in Week 1 (*p* = 0.031) and Weeks 3–4 (both *p* < 0.001), with no significant difference in Week 2. Thrombocytopenia rates were similar between the 14d and > 14d cohorts across all weekly intervals (Figure 3B). In the Always‐7d sensitivity analysis, thrombocytopenia did not differ between Always 7d and > 14d in any weekly interval (all *p* ≥ 0.176) and differed from 14d only in Week 4 (*p* = 0.023).

Grade 4 neutropenia (ANC < 0.5 × 10^9^/L) was also less frequent with 7d than with either 14d or > 14d across Weeks 1–4, with all comparisons reaching statistical significance (7d vs. 14d: all *p* < 0.01; 7d vs. > 14d: all *p* < 0.05). Comparisons between 14d and > 14d were not significant in Weeks 1–3 but differed in Week 4 (*p* < 0.001; Figure 3C). In the Always‐7d sensitivity analysis, the rate of grade 4 neutropenia remained lower with Always 7d than with 14d across Weeks 1–4 (*p* < 0.001, *p* = 0.002, *p* = 0.013 and *p* < 0.001, respectively) and lower than with > 14d in Weeks 2–4 (*p* < 0.001, *p* = 0.035 and *p* < 0.001), but not in Week 1 (*p* = 0.190). Detailed results of the strict Always‐7d safety sensitivity analysis are provided in Tables S1A–C.

A greater proportion of cycles required at least one platelet transfusion in the > 14d cohort than in the 7d cohort (53.4% vs. 25.8%; *p* = 0.02), whereas the mean number of platelet units per cycle did not differ significantly (3.90 vs. 1.89; *p* = 0.07). The proportion of cycles requiring at least one RBC transfusion was also numerically higher in the > 14d cohort than in the 7d cohort (69.9% vs. 55.7%; *p* = 0.06), as was the mean number of RBC units per cycle (4.76 vs. 2.78; *p* = 0.07), although these differences did not reach statistical significance. No significant differences were observed between the 14d and 7d cohorts for platelet or RBC transfusion requirements. In the sensitivity analysis, Always 7d showed a non‐significant lower proportion of required platelet transfusion per cycle compared with the > 14d cohort (43.9% vs. 30.2%; *p* = 0.29).

### 3.4 Survival Differences Persist After Adjustment for Baseline Imbalance and Time‐Varying Neutropenia Burden

In univariable Cox analyses, prior HMA therapy (HR 2.04, 95% CI 1.06–3.93; *p* = 0.03) and non‐favourable ELN 2024 risk (HR 2.43, 95% CI 1.28–4.61; *p* < 0.01) were associated with inferior OS (Table 2). ECOG 2–3 showed borderline significance (HR 1.87, 95% CI 0.99–3.55; *p* = 0.054). Baseline ANC < 0.8 × 10^9^/L was associated with a lower hazard of death (HR 0.44, 95% CI 0.22–0.86; *p* = 0.02). Other baseline covariates were not significantly associated with OS.

**TABLE 2:** Univariable and multivariable Cox regression analyses for overall survival.

Variables	*N*	Events	HR (95% CI), univariate	*p* (univariate)	HR (95% CI), multivariate	*p* (multivariate)
ANC < 0.8 × 10^9^/L	61	40	0.44 (0.22–0.86)	**0.017**		
BM blasts (per 10%)	61	40	1.06 (0.96–1.16)	0.277		
ELN 2024 non‐favourable	61	40	2.43 (1.28–4.61)	**0.006**	3.22 (1.49–7.00)	**0.003**
Haemoglobin ≤ 100 g/L	61	40	1.27 (0.56–2.88)	0.565		
ECOG 2–3	61	40	1.87 (0.99–3.55)	0.054		
Platelets < 50	61	40	1.71 (0.91–3.21)	0.096		
14d VEN schedule	61	40	0.79 (0.39–1.59)	0.504	0.95 (0.39–2.31)	0.905
7d VEN schedule	61	40	0.46 (0.18–1.14)	0.093	0.33 (0.13–0.87)	**0.025**
Age ≥ 75 years	61	40	1.61 (0.86–3.02)	0.139	2.16 (1.12–4.19)	**0.022**
Non‐diploid karyotype	61	40	1.02 (0.55–1.89)	0.962		
Prior HMA therapy	61	40	2.04 (1.06–3.93)	**0.034**	2.37 (1.16–4.84)	**0.018**

*Note:* Cox proportional hazards models for overall survival (*n* = 61 patients; 40 events). Univariable models were fitted for each listed covariate. The multivariable model included VEN schedule, prior HMA therapy, ELN 2024 non‐favourable risk and age ≥ 75 years. The > 14d VEN schedule was used as the reference category for schedule comparisons. Variables without multivariable estimates were assessed in univariable analysis only. Bold values indicate statistical significance *p* < 0.05.

Abbreviations: 7d, 7 days; 14d, 14 days; ANC, absolute neutrophil count; BM, bone marrow; ECOG, Eastern Cooperative Oncology Group; ELN, European LeukemiaNet; HMA, hypomethylating agent; HR, hazard ratio; VEN, venetoclax.

Given baseline imbalances between the 7d and > 14d groups, adjusted Cox models included age ≥ 75 years, prior HMA therapy and ELN 2024 risk in addition to the VEN schedule. In this multivariable model (*n* = 61; 40 events), the 7d schedule remained associated with improved OS compared with > 14d (HR 0.33, 95% CI 0.13–0.87; *p* = 0.03), whereas the 14d schedule did not differ from > 14d. Prior HMA therapy (HR 2.37, 95% CI 1.16–4.84; *p* = 0.02), non‐favourable ELN 2024 risk (HR 3.22, 95% CI 1.49–7.00; *p* < 0.01) and age ≥ 75 years (HR 2.16, 95% CI 1.12–4.19; *p* = 0.02) remained independently associated with inferior OS.

To account for time‐varying neutropenia, we fitted a time‐dependent Cox model incorporating rolling 30‐day grade 4 neutropenia burden. Higher recent neutropenia burden was strongly associated with inferior OS. Expressed on a clinically interpretable scale, each additional 7 days of grade 4 neutropenia during the preceding 30 days was associated with a 48% higher hazard of death (HR 1.48, 95% CI 1.19–1.84; *p* < 0.001; Table 3). After adjustment for neutropenia burden, the association between the 7d schedule and lower mortality hazard versus > 14d persisted (HR 0.34, 95% CI 0.13–0.87; *p* = 0.03), whereas no difference was observed between 14d and > 14d.

**TABLE 3:** Time‐dependent Cox regression analysis incorporating rolling 30‐day grade 4 neutropenia burden.

Variables	HR (95% CI)	*p*
14d VEN schedule vs. > 14d	0.77 (0.30–1.97)	0.586
7d VEN schedule vs. > 14d	0.34 (0.13–0.87)	**0.025**
Prior HMA therapy	2.88 (1.44–5.76)	**0.003**
ELN 2024 non‐favourable	2.68 (1.22–5.91)	**0.014**
Age ≥ 75 years	2.13 (1.11–4.12)	**0.024**
Rolling 30‐day grade 4 neutropenia burden (per additional 7d)	1.48 (1.19–1.84)	**< 0.001**

*Note:* Time‐dependent Cox proportional hazards model including rolling 30‐day grade 4 neutropenia burden, defined as the proportion of the preceding 30 days spent with ANC < 0.5 × 10^9^/L and rescaled to reflect the hazard ratio per additional 7 days of grade 4 neutropenia within the preceding 30‐day window. Based on 61 patients (40 events) and 4253 patient‐time intervals derived from the start‐stop dataset for time‐dependent Cox modelling. Robust variance estimation clustered by patient. Reference category for VEN schedule: > 14d. Bold values indicate statistical significance *p* < 0.05.

Abbreviations: 7d, 7 days; 14d, 14 days; ANC, absolute neutrophil count; CI, confidence interval; ELN, European LeukemiaNet; HMA, hypomethylating agent; HR, hazard ratio; OS, overall survival; VEN, venetoclax.

## 4 Discussion

In this retrospective single‐centre cohort, abbreviated VEN exposure of 7 days in combination with AZA or DEC was associated with reduced treatment‐related toxicity and did not appear to compromise remission quality. Although unadjusted OS did not differ significantly across schedules, the 7d cohort showed numerically longer median OS, fewer infections, more complication‐free cycles, lower rates of grade 4 cytopenias and lower platelet transfusion requirements than the longer‐exposure cohorts. In adjusted analyses, the 7d schedule remained associated with improved OS compared with > 14d exposure, whereas the 14d schedule did not.

These findings align with emerging real‐world data suggesting that shorter VEN schedules may preserve efficacy while improving tolerability. Willekens et al. reported comparable remission and survival outcomes with 7d VEN exposure together with reduced early mortality and platelet transfusion requirements compared with standard 28‐day exposure [8]. Likewise, Wurm et al. observed similar response outcomes across shorter and longer VEN exposure groups at first bone marrow assessment [14]. Our study extends these observations by providing a longitudinal cycle‐level analysis of complications, cytopenias and transfusion burden across VEN schedules. This is clinically relevant because, in routine practice, the feasibility of continued HMA‐VEN therapy is often determined less by initial remission induction than by recurrent infectious and haematological toxicity over successive cycles.

The 7d VEN schedule resulted in the greatest temporal overlap between VEN and HMA exposure, whereas longer schedules included additional VEN‐only days after HMA completion. This may be biologically relevant, as AZA has been shown to induce NOXA/PMAIP1 and prime AML cells for VEN‐mediated apoptosis [15, 16]. Concentrating VEN exposure within this HMA‐primed window could therefore preserve relevant VEN‐HMA synergy while limiting prolonged VEN‐only exposure and associated myelosuppression. Prospective studies with pharmacodynamic and molecular correlates are needed to define the optimal duration and timing of VEN exposure relative to HMA administration.

The survival analyses should be interpreted in the context of non‐random treatment assignment. The 7d cohort was older and more frequently had impaired performance status, whereas the > 14d cohort had higher marrow blast percentages and a greater proportion of ELN 2024 non‐favourable disease. These bidirectional imbalances make crude comparisons difficult and support the use of adjusted models. In the multivariable Cox model, prior HMA exposure, non‐favourable ELN 2024 risk and age ≥ 75 years were independently associated with inferior OS, while the 7d schedule remained associated with better OS than > 14d. This does not prove causality, but it argues against the observed differences being explained solely by more favourable baseline risk in the 7d cohort. To further address potential time‐dependent treatment‐selection bias, we performed a strict Always‐7d sensitivity analysis separating patients who received 7 days of VEN in all documented cycles from longer‐schedule groups. Although limited by small numbers, this analysis showed no adverse OS signal for the Always‐7d group, with numerically longer median OS and non‐significant pairwise comparisons versus both longer‐schedule groups. Consistent with the main toxicity analysis, grade 4 neutropenia burden and infection‐associated cycles were lower in the Always‐7d group, whereas thrombocytopenia findings were less consistent. These sensitivity analyses support, but do not prove, that the favourable outcomes in the 7d cohort were not solely attributable to later de‐escalation after prolonged response. A further strength of the analysis is the incorporation of time‐varying grade 4 neutropenia burden. Recent severe neutropenia was strongly associated with inferior OS, even after accounting for treatment schedule and baseline covariates. Importantly, adjustment for neutropenia burden did not materially alter the association between the 7d schedule and improved OS versus > 14d. This suggests that reduced neutropenia alone is unlikely to explain the apparent survival advantage of abbreviated VEN exposure fully. More broadly, shorter exposure may improve outcomes through a combination of lower infectious morbidity, reduced cumulative haematological toxicity and better treatment continuity. We did not perform an analogous rolling thrombocytopenia model because platelet counts in this setting are directly modified by transfusion support and are therefore less readily interpretable as a biological time‐varying exposure.

Several limitations should be acknowledged. First, this was a retrospective analysis with a limited sample size, so statistical power was modest and residual confounding cannot be excluded. Importantly, baseline characteristics were not fully balanced between treatment cohorts, including a significant difference in baseline bone marrow blast percentage, which may reflect differences in disease burden and could have influenced response and survival outcomes. Second, VEN schedule assignment was physician‐directed and could change over time, including switches from longer schedules to 7 days, introducing potential survivorship and selection bias. Third, the cohort was clinically heterogeneous, including both frontline‐ and later‐line‐treated patients, as well as treatment with either AZA or DEC and variable azole co‐medication requiring VEN dose adjustment. Fourth, the comparator group was defined as > 14d exposure rather than a uniform 28‐day schedule, which limits direct extrapolation to the conventional full‐length regimen. Finally, the cycle‐level toxicity analyses are informative for treatment burden but are not independent across cycles and should therefore be interpreted descriptively.

Taken together, our data support the clinical feasibility of abbreviated VEN exposure in patients with AML who are older, frail or otherwise unsuitable for intensive therapy. A 7d schedule was associated with lower toxicity and preserved response quality, and in adjusted analyses, also with improved OS compared with > 14d exposure. These findings support the concept that shorter VEN schedules may represent a pragmatic strategy to improve tolerability without obvious loss of anti‐leukaemic activity, but prospective validation is required before firm practice recommendations can be made.

## Author Contributions

L.S., L.S.S.‐B. and A.S. conceived the study, oversaw the project and wrote the manuscript. M.G., M.A., G.M., S.Kl. and S.Kr. treated patients. L.S. and L.S.S.‐B. collected the data, while C.G. collected the laboratory data. L.S., L.S.S.‐B. and A.S. analysed and interpreted the data. Interpretation of data: All authors. Involved in drafting/revising critically for intellectual content: L.S., L.S.S.‐B., A.F., S.Kl., N.S., P.W., W.‐K.H. and A.S. All authors approved the final version of the manuscript.

## Funding

Funding was provided by the Medical Faculty Mannheim, Heidelberg University and Torsten Haferlach Leukemia Diagnostics Foundation.

## Ethics Statement

All clinical investigations were conducted in accordance with the principles of the Declaration of Helsinki.

## Consent

All patients had provided written consent permitting the use of their anonymised clinical data for research purposes.

## Conflicts of Interest

The authors declare no conflicts of interest.

## Supporting information


**Figure S1:** Post‐remission overall survival by predominant post‐remission venetoclax schedule. Kaplan–Meier curves show overall survival measured from first remission among responders with documented post‐remission VEN‐containing treatment cycles. Patients were stratified according to their predominant post‐remission VEN schedule: > 14 days (*n* = 12), 14 days (*n* = 8) or 7 days (*n* = 9). Numbers at risk are shown below the plot.
**Table S1A:** Cycle‐level complications in the strict Always‐7d sensitivity analysis. Cycle‐level complication outcomes are shown for patients receiving 7 days of venetoclax in all documented cycles compared with longer‐schedule groups. Outcomes are reported per VEN‐containing treatment cycle. Pairwise *p* values compare the Always‐7d group with each longer‐schedule group. VEN, venetoclax.
**Table S1B:** Grade 4 neutropenia by cycle week in the strict Always‐7d sensitivity analysis. Grade 4 neutropenia was defined as absolute neutrophil count < 0.5 × 10^9^/L and is shown by weekly cycle interval among available laboratory measurements. Pairwise *p* values compare the Always‐7 days group with each longer‐schedule group.
**Table S1C:** Grade 4 thrombocytopenia by cycle week in the strict Always‐7d sensitivity analysis. Grade 4 thrombocytopenia was defined as platelet count < 25 × 10^9^/L and is shown by weekly cycle interval among available laboratory measurements. Pairwise *p* values compare the Always‐7d group with each longer‐schedule group.

## Data Availability

The data that support the findings of this study are available from the corresponding author upon reasonable request.
