# Supplementary material for: Dose‐Adjusted Treatment With 7‐Day Venetoclax Plus Azacitidine or Decitabine in Patients With Acute Myeloid Leukaemia
Source: Eur J Haematol. 2026 Jul 30;117(5):1358–68. doi: 10.1111/ejh.70279 (PMC13643631; doi:10.1111/ejh.70279)
Supplement: Supplementary file 1 [file EJH-117-1358-s001.docx]

**Supplement**

**Supplementary Figures**

**Supplementary Figure S1. Post-remission overall survival by predominant post-remission venetoclax schedule.** Kaplan-Meier curves show overall survival measured from first remission among responders with documented post-remission VEN-containing treatment cycles. Patients were stratified according to their predominant post-remission VEN schedule: >14 days (n=12), 14 days (n=8), or 7 days (n=9). Numbers at risk are shown below the plot.

**Supplementary Tables**

**Supplementary Table S1A. Cycle-level complications in the strict Always-7d sensitivity analysis.** Cycle-level complication outcomes are shown for patients receiving 7 days of venetoclax in all documented cycles compared with longer-schedule groups. Outcomes are reported per VEN-containing treatment cycle. Pairwise p-values compare the Always-7d group with each longer-schedule group. Abbreviations: VEN, venetoclax.

| Outcome | >14d group | 14d group | Always 7d | Always 7d vs >14d | Always 7d vs 14d |
| --- | --- | --- | --- | --- | --- |
| Total VEN-containing cycles | 144 | 72 | 43 | — | — |
| Infection-associated cycles | 47/144 (32.6%) | 21/72 (29.2%) | 4/43 (9.3%) | p=0.003 | p=0.012 |
| Complication-free cycles | 90/144 (62.5%) | 49/72 (68.1%) | 38/43 (88.4%) | p=0.001 | p=0.014 |

**Supplementary Table S1B. Grade 4 neutropenia by cycle week in the strict Always-7d sensitivity analysis.** Grade 4 neutropenia was defined as absolute neutrophil count <0.5×10⁹/L and is shown by weekly cycle interval among available laboratory measurements. Pairwise p-values compare the Always-7d group with each longer-schedule group.

| Weekly interval | >14d group | 14d group | Always 7d | Always 7d vs >14d | Always 7d vs 14d |
| --- | --- | --- | --- | --- | --- |
| Days 0-7 | 148/463 (32.0%) | 108/237 (45.6%) | 25/101 (24.8%) | p=0.190 | p<0.001 |
| Days 8-14 | 129/229 (56.3%) | 64/123 (52.0%) | 8/35 (22.9%) | p<0.001 | p=0.002 |
| Days 15-21 | 138/218 (63.3%) | 73/107 (68.2%) | 11/27 (40.7%) | p=0.035 | p=0.013 |
| Days 22-28 | 129/185 (69.7%) | 65/83 (78.3%) | 19/45 (42.2%) | p<0.001 | p<0.001 |

**Supplementary Table S1C. Grade 4 thrombocytopenia by cycle week in the strict Always-7d sensitivity analysis.** Grade 4 thrombocytopenia was defined as platelet count <25×10⁹/L and is shown by weekly cycle interval among available laboratory measurements. Pairwise p-values compare the Always-7d group with each longer-schedule group.

| Weekly interval | >14d group | 14d group | Always 7d | Always 7d vs >14d | Always 7d vs 14d |
| --- | --- | --- | --- | --- | --- |
| Days 0-7 | 134/567 (23.6%) | 84/280 (30.0%) | 30/115 (26.1%) | p=0.632 | p=0.465 |
| Days 8-14 | 116/314 (36.9%) | 77/166 (46.4%) | 26/55 (47.3%) | p=0.176 | p=1.000 |
| Days 15-21 | 93/295 (31.5%) | 69/153 (45.1%) | 18/50 (36.0%) | p=0.518 | p=0.324 |
| Days 22-28 | 58/283 (20.5%) | 48/121 (39.7%) | 15/67 (22.4%) | p=0.739 | p=0.023 |
